# Ethnobotanical Survey of Medicinal Plants Used in the Treatment of COVID-19 and Related Respiratory Infections in Ogbomosho South and North Local Government Areas, Oyo State, Nigeria

**DOI:** 10.3390/plants11192667

**Published:** 2022-10-10

**Authors:** Christiana Adeyinka Odebunmi, Tomi Lois Adetunji, Ademola Emmanuel Adetunji, Ahmed Olatunde, Oluwatosin Esther Oluwole, Idowu Ayodeji Adewale, Abdulrasak Opeyemi Ejiwumi, Chinwenwa Esther Iheme, Taiwo Opeyemi Aremu

**Affiliations:** 1 Derived Guinea Savannah Research Station, Forestry Research Institute of Nigeria, Oke-Aduin, Ogbomosho P.O. Box 164, Nigeria; 2 Unit for Environmental Sciences and Management (UESM), Faculty of Natural and Agricultural Sciences, North-West University, Potchefstroom 2520, South Africa; 3 Department of Molecular and Cell Biology, University of Cape Town, Cape Town 7701, South Africa; 4 Department of Medical Biochemistry, Abubakar Tafawa Balewa University, Bauchi 740272, Nigeria; 5 Division of Epidemiology & Community Health, School of Public Health, University of Minnesota, 1300 S. 2nd Street, Minneapolis, MN 55455, USA; 6 Department of Medical Microbiology, University of Ilorin Teaching Hospital, Old Jebba Road, Oke Ose, Ilorin 240001, Nigeria; 7 Department of Health Services Management and Policy, College of Public Health, East Tennessee State University, 3rd Floor Sherrod Library, Johnson City, TN 37614, USA; 8 Division of Environmental Health Sciences, School of Public Health, University of Minnesota, 420 Delaware Street SE, Minneapolis, MN 55455, USA; 9 Department of Pharmaceutical Care & Health Systems (PCHS), College of Pharmacy, University of Minnesota, 308 Harvard Street SE, Minneapolis, MN 55455, USA

**Keywords:** antiviral activity, drug development, ethnobotanical indices, immunomodulatory, traditional African medicine

## Abstract

Coronavirus disease 2019 (COVID-19) has extensively spread worldwide with high mortality. Besides vaccination, the United States Food and Drug Administration approved only one oral medication as a treatment. Medicinal plants with antiviral and immunomodulatory properties could be explored as complementary treatments for COVID-19. Ogbomosho is home to such plants traditionally used to treat infectious diseases in Nigeria, making it relevant in complementary medicine. An ethnobotanical survey of medicinal plants used to treat COVID-19 and related ailments, including cough and flu in Ogbomosho South and North Local Government Areas, Nigeria, was conducted using a semi-structured questionnaire. Information was obtained from 56 participants, consisting of different groups of individuals with native knowledge of medicinal plants, and ethnobotanical indices, including the frequency of citation (FC), relative frequency of citation (RFC), and fidelity level (FL) were computed. Twenty-six medicinal plants (17 families) were used to treat COVID-19, 31 (20 families) for cough, and 29 (19 families) for flu. The most cited plant was *Zingiber officinale* (FC = 10; RFC = 0.18; FL = 18%) for treating COVID-19, *Citrus limon* (FC = 13; RFC = 0.23; FL = 23%) for cough, and *Zingiber officinale* (FC = 9; RFC = 0.16; FL = 16%) for flu. Leaves were the most used plant part for treating COVID-19 and flu, while the bark was the most used for cough. Trees and herbs were the most cited plant growth forms. The herbal remedies were mostly prepared by decoction and infusion and were mainly administered orally. Further research should be conducted on the identified species for the scientific validation of their antiviral and immunomodulatory efficacies and safety for use.

## 1. Introduction

Upper respiratory tract infections (URTIs) are one of the most common diseases in primary care globally [1]. Mainly caused by viruses, the most commonly occurring symptoms of URTIs are sore throat, nasal congestion, runny nose, cough, headache, etc. Whilst these conditions are not usually fatal, the symptoms may significantly affect human efficiency and quality of life [1]. Some poorly managed URTIs can result in complications, including myocarditis, pneumonia, otitis media, etc., accounting for significant morbidity and mortality globally [2]. In December 2019, a novel virus known as the Severe Acute Respiratory Syndrome Coronavirus-2 (SARS-CoV-2), which causes respiratory illness, emerged in Wuhan, China, and has spread to almost all parts of the world [3]. As a result of its massive spread globally, the World Health Organization (WHO) declared the coronavirus disease 2019 (COVID-19) a pandemic on the 11 March 2020 [4,5]. Common symptoms of COVID-19 include headache, fever, fatigue, malaise, and dry cough, while less commonly occurring symptoms include sore throat, nausea, diarrhea, generalized body pain, discoloration of fingers and toes, conjunctivitis, and loss of smell or taste [3,6]. In severe cases, COVID-19 results in life-threatening symptoms such as difficulty in breathing, loss of speech or movement, chest pain, shortness of breath and pressure, typical of acute respiratory distress syndrome (ARDS) [3,7]. The WHO reported 14.9 million deaths linked to the COVID-19 pandemic globally between 1 January 2020 and 31 December 2021 [8].

The Nigerian Federal Ministry of Health confirmed the first national case of COVID-19 in Lagos State on 27 February 2020. Over 256,000 infections have been recorded since the pandemic began while over 3000 COVID-19-related deaths have been reported, making Nigeria one of the 20 countries accounting for over 80% of global mortality resulting from COVID-19 from January 2020 to December 2021 [8].

The increasing spread and mortality resulting from COVID-19 across the globe have led to different interventions, including personal hygiene, quarantine, isolation, and the development of vaccines, to control the spread of the virus [9]. Although an oral medication, Paxlovid, has been developed to treat mild to moderate COVID-19 in vulnerable and severely ill individuals aged 12 and older (weighing at least 40 kg), no other evidence-based oral drugs have been developed to prevent COVID-19 infection. While there may be limited access to Paxlovid in low- and middle-income countries, no evidence-based and cost-saving medications have been adapted for treating or preventing COVID-19 in people living in low-resource countries [10,11]. Besides, no vaccine has been identified to be 100 percent effective against the virus and its emerging variants. Hence, scientists have searched more for safe natural products with antiviral and immunomodulatory activities that can serve as potential prophylactic complementary and/or alternative therapeutics for COVID-19 [12,13].

For ages, medicinal plants have been used by different people worldwide as traditional medical treatments and for the prevention of various ailments, including acute respiratory infections [14]. Medicinal plant use has been recognized in some countries (e.g., Uganda, Ghana, Tanzania, etc.) as the leading primary care medication, and over 80% of people in low and middle-income countries depend directly on it [15,16,17]. Medicinal plants are widely used due to their availability, accessibility, affordability, and perceived safety relative to modern medicine [18]. In many African countries, including Nigeria, herbal medicine is well embraced, as up to 90% of rural dwellers rely on it for their primary health care [16,19]. The efficacy of medicinal plants is attributed to the presence of bioactive compounds that confer several bioactivities with therapeutic properties, such as antiviral, antimicrobial, anti-inflammatory, and immunomodulatory activities, etc. [12].

Since the emergence of COVID-19, the use and demand for some medicinal plants has increased worldwide, particularly those used to treat symptoms (for example, sore throat, flu, and cough) associated with COVID-19 [20]. Ethnobotanical surveys focus on multiplex links between local plants and inhabitants, including cultural beliefs and practices linked to several forms of application [21,22]. These surveys are vital in expressing the importance of plant species, that is, for discovering new therapeutic agents [23]. An ethnobotanical survey of medicinal plants is thus applicable as a vital approach for identifying and selecting plants that can be subjected to further phytochemical and pharmacological screening for developing novel therapeutic agents [24].

In Nigeria, the Yoruba people comprise about 40% of the total population and are largely distributed in the southwestern states (Oyo, Ondo, Osun, Ekiti, Ogun, and Lagos) of Nigeria [25,26]. The southwestern zone of Nigeria is a humid tropical area with rainy and dry seasons; hence, the states in this zone have rich floristic diversity and cultural heritage [25,26]. The major cities and towns in the southwestern states include Ogbomosho, Abeokuta, Ibadan, Ede, Ikire, Osogbo, Ile-Ife, and Lagos [25,26]. The major traditional occupations of the inhabitants of this zone are fishing, pottery, farming, indigenous medical practices, and blacksmithing [25]. The recent study of Ajao et al. [26], which compiled the list of angiosperms in the southwestern zone of Nigeria, identified a total of 493 angiosperm species belonging to 99 families, out of which 418 species are utilized for traditional medicinal purposes. Despite the rich floristic diversity of medicinal species in this zone, studies focusing on the indigenous use and traditional medicinal plant applications are limited [26]. Ogbomosho, one of the major towns in the south-western zone of Nigeria, is endowed with a wide variety of indigenous medicinal plants that local herbalists commonly utilize to treat several ailments, including infectious diseases [27]. The present study aimed to document and provide basic knowledge of medicinal plants used by herb sellers, traditional health practitioners, and other people with indigenous knowledge in the treatment of COVID-19, cough, and flu in Ogbomosho South and North Local Government in Ogbomosho, Oyo State, Nigeria. The ethnobotanical survey of medicinal plants used in the management of COVID-19 in Ogbomosho Local Government Areas of Oyo State, Nigeria, will provide information on the availability, classification, preparation, and therapeutic potential of medicinal plants used in the region for treating COVID-19 and related respiratory ailments, particularly cough and flu.

## 2. Materials and Methods

### 2.1. Study Area

**Figure 1:**
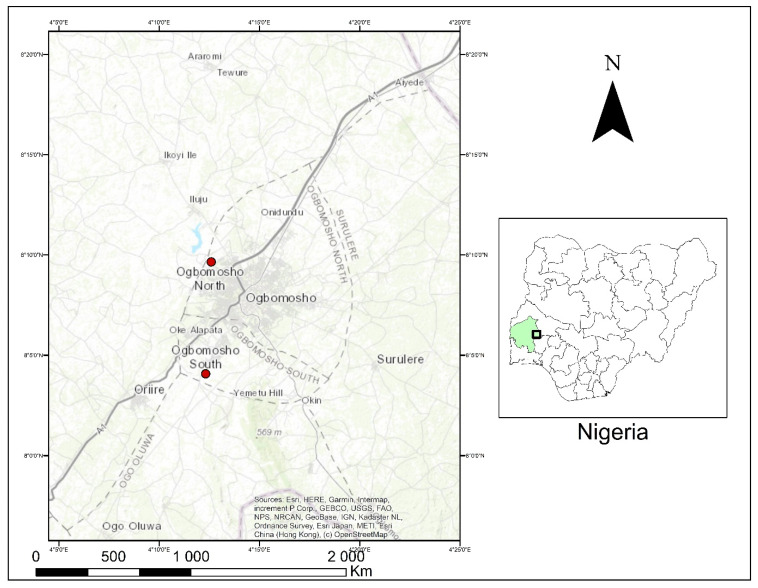
Ogbomosho North and South Local Government Areas, Oyo State, Nigeria.

The study was conducted between October 2021 and March 2022 in Ogbomosho North and Ogbomosho South Local Government Areas of Oyo State, Nigeria (Figure 1). Ogbomosho lies between latitude 8′08′00″ N and longitude 4′16′00″ E, with an approximate population density of 454,690 [28]. The city is inhabited primarily by the Yoruba ethnic group, and agriculture is the major economy.

### 2.2. Collection of Data

Ethnobotanical information was obtained from participants using a semi-structured questionnaire. The participants were purposively selected, comprising herb sellers, traditional health practitioners, farmers, and individuals with indigenous knowledge. The questionnaire was prepared in English. However, to facilitate efficient communication, informal discussion with herb sellers and traditional health practitioners was done in Yoruba, the local language in these local government areas. A total of 56 participants were interviewed, and the participants were interviewed individually to maintain confidentiality. Data collected included age, gender, occupation, educational background, residence, local names of the plants used in treating COVID-19 and related ailments, plant part used, method of preparation/extraction, and administration method. The study and data collection were done following the regulations made by the International Society of Ethnobiology (available at https://www.ethnobiology.net, accessed on 30 September 2022).

### 2.3. Collection and Identification of Medicinal Plants

Plant collection was done with the help of herb sellers and traditional health practitioners, using the plants’ local names (in the Yoruba language). The scientific identification of plants was made at IFE herbarium of the Obafemi Awolowo University, Ile-Ife, Osun State, Nigeria (herbarium code IFE, according to Thiers [29]), and voucher specimens were prepared and deposited at the herbarium. In addition, the currently accepted names of all plants identified were verified on “World Flora Online” (www.worldfloraonline.org, accessed on 1 August 2022).

### 2.4. Data Analysis

Descriptive statistics were carried out on the participants’ demographic information. The data obtained from the documented plants were analyzed using three ethnobotanical indices: frequency of citation (FC), the relative frequency of citation (RFC), and fidelity level (FL).

Following the methods described by Tardio and Pardo-de-santayana [30], RFC was calculated as:

RCF = FC/N

where FC = Frequency of citation/mention, and N = number of participants in the survey.

Fidelity level was calculated as described by Friedman et al. [31]:

FL = (Ip/Iu) × 100

where Ip = Number of respondents that mentioned a particular plant species used to treat an ailment being considered, and

Iu = Total number of respondents in the survey.

### 2.5. Ethical Approval

The study was approved by the Committee of Ethics of the Forestry Research Institute of Nigeria, Ibadan, Nigeria, with the ethical approval number CFGO711FRIN06. Informed consent was obtained from the participants prior to data collection.

## 3. Results and Discussion

### 3.1. Demographic Information

The interview started with an assessment of the demographic characteristics of the participants (Table 1). Fifty-six participants in the age range of 20 to 61 with indigenous knowledge of medicinal plants were interviewed. Most participants were female (58.9%). Individuals aged 51 and above accounted for about 55.4% of the participants. There were more individuals with primary education, constituting about 46.4% of the participants, followed by individuals with secondary education (33.9%). Most of the participants (48.2%) were herb sellers. The participants who all lived in rural areas were either Yoruba (96.4%) or Igbo (3.6%). It is known that variations in gender, age, and educational status of participants have an important relationship with ethnomedicinal knowledge [32].

The predominance of the female gender in the present study suggests that women have more knowledge of indigenous medicinal plants than their male counterparts in the study area. The current results corroborate the findings of Chukwuma et al. [33], which reported the dominance of female participants with herbal knowledge in an ethnobotanical survey done in Ado-Ekiti, a Southwestern State in Nigeria. Similarly, women were the most involved and informed in the knowledge and utilization of medicinal plants used for preventing and treating COVID-19, for example, in Algeria [32]. Furthermore, the study conducted by Teixidor-Toneu et al. [34] indicated that women have three-fold more knowledge of medicinal plants than men in Morocco. This was attributed to the frequency of cooking by women (since most medicinal plants are widely used in food preparation), women’s specific conditions, and gender-specific culture. In most African countries, rural areas historically collected different native plants’ parts for their diets and family health needs [35].

Generally, the majority (55.4%) of the people with indigenous knowledge in this study were older than age 51. This result is similar to previous reports on the ethnobotanical survey of indigenous medicinal plants in Nigeria [24] and Northern Morocco [36]. The predominance of elderly participants with indigenous knowledge in the present study agrees with previous reports that older people (above age 50) are usually more knowledgeable about the practice of herbal medicine [24]. The results also revealed a gap between the older and younger generations in the knowledge of indigenous plant use in the study area. The erosion of the knowledge of medicinal plant among younger generations has been reported in other parts of the world. For example, in Western and Northwestern Himalaya, the loss of knowledge regarding the use of medicinal plants in the younger generation was reported [37,38]. This valuable knowledge of medicinal plants and their uses erodes in the younger generation is largely due to westernization, higher education level, and habitat destruction of medicinal plants [34,37,38]. Therefore, it is important that indigenous knowledge is documented, preserved, shared with the younger generations, and that plant resources are conserved.

Regarding the level of education, individuals with a tertiary education level constituted the minority (19.64%) of the participants. This result may be attributed to the fact that the practice of traditional medicine or herbalism does not require a degree but is acquired by experience and learning from older generations [36]. Our result is in line with an ethnobotanical survey of local flora used for medicinal purposes conducted in Lagos, Nigeria, which reported that 79% of the participants with indigenous knowledge had only a primary education [39].

### 3.2. Medicinal Plants Recorded

**Figure 2:**
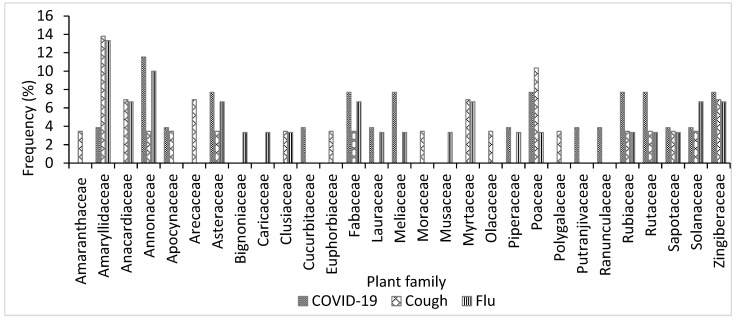
Frequency of plant families used for treating COVID-19, cough, and flu in Ogbomosho North and South Local Government Areas, Oyo State, Nigeria.

In total, 26 medicinal plant species belonging to 17 families were used to treat COVID-19, 31 medicinal plants from 20 families were used to treat cough, and 29 plant species belonging to 19 families were used to treat flu (Table 2, Table 3 and Table 4). For COVID-19 treatment, the most represented families in terms of the number of species were Annonaceae, Meliaceae, Rubiaceae, Asteraceae, Zingiberaceae, Rutaceae, and Fabaceae, with 2–3 species in each family. The most representative families for treating cough were the Amaryllidaceae, Poaceae, Zingiberaceae, Anacardiaceae, Rutaceae, Myrtaceae, Fabaceae, and Arecaceae, with 2–3 species per family. For flu-related symptoms, the most representative families were the Annonaceae (three species), followed by Amaryllidaceae, Zingiberaceae, Compositae, Solanaceae, Myrtaceae, and Fabaceae, with two species each (Figure 2). This result is similar to that reported by Benkhaira et al. [36], where Asteraceae and Zingiberaceae were listed as some of the most representative medicinal plant families used for treating and preventing COVID-19 in Northern Morocco. It is also similar to the findings of Lawal et al. [40], where Fabaceae and Poaceae were listed as some of the most represented plant families used for treating coughs in Osun state, Nigeria. The relatively high number of plants and families identified in this study for the treatment of COVID-19, cough, and flu indicates the richness and diversity of the Nigerian flora for various therapeutic purposes. The diverse phytochemicals present in these florae, which are relatively unexplored, can serve as a potential source of drug development for treating different respiratory ailments [41].

### 3.3. Plant Parts and Growth Forms of Medicinal Plants Used for Treating COVID-19, Cough, and Flu

**Figure 3:**
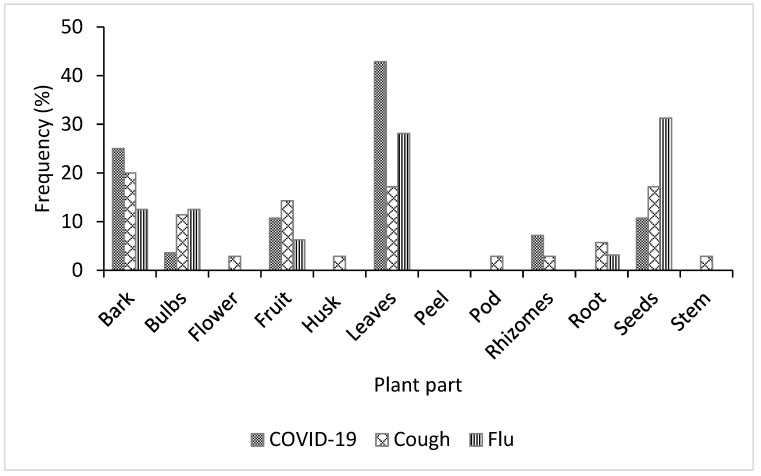
Frequency of plant parts used for treating COVID-19, cough, and flu in Ogbomosho North and South Local Government Areas, Oyo State, Nigeria.

As participants in the study area reported, diverse plant parts such as bark, bulbs, flower, husk, leaves, rhizomes, and root were used for treating COVID-19, cough, and flu (Figure 3). However, leaves were the most commonly used plant part for treating COVID-19 (39%) and flu (31%), while the bark (20%) was the most commonly used part for treating cough (Figure 3). Several previous studies in Nigeria have also reported that leaves and bark were more utilized in the traditional treatment of different ailments than other plant parts [24,39,40]. The widespread use of leaves for herbal medicine preparation may be attributed to their relatively high abundance, accessibility, and ease of collection [42]. Additionally, the relatively higher photosynthetic and metabolic activities occurring in the aerial parts of most plants (particularly leaves) may have contributed to the build-up of bioactive substances with therapeutic properties [42]. 

**Figure 4:**
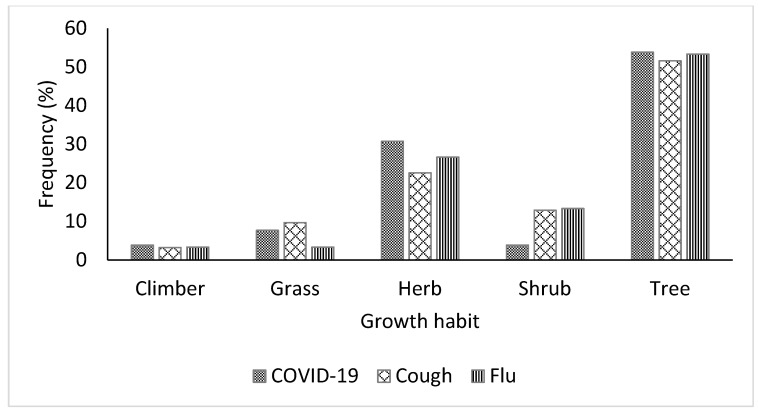
Growth forms of medicinal plants used for the treatment of COVID-19, cough, and flu in Ogbomosho North and South Local Government Areas, Oyo State, Nigeria.

In the study area, the herbal remedies used to treat and prevent COVID-19, cough, and flu were sourced from different growth forms, including trees, shrubs, herbs, grasses, and climbers (Figure 4). However, for the three respiratory infections referenced, a significant number of the medicinal plants were trees (54%, 52%, and 53% for COVID-19, cough, and flu, respectively), followed by herbs (31%, 23%, and 27% for COVID-19, cough, and flu, respectively), and climbers were the lowest growth forms utilized for treating COVID-19, cough, and flu. In terms of sustainability and conservation, harvesting leaves from trees may be more sustainable than other growth forms, given that trees are more resilient due to their sizes [24]. The dominance of woody perennials as sources of herbal remedies may be connected to the rainforest vegetation of the study area. Previous studies [24,43] have reported similar dominance of woody plants in ethnobotanical surveys of plants conducted in the same rainforest zones in Nigeria.

### 3.4. Method of Preparation and Mode of Administration of Medicinal Plants Species Used for Treating COVID-19, Cough, and Flu

**Figure 5:**
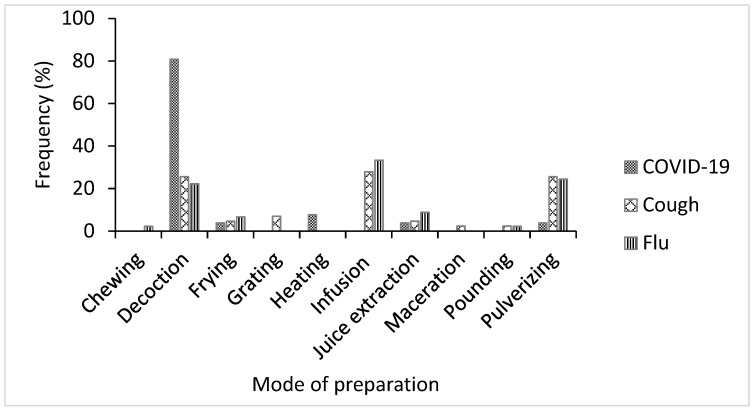
Methods of preparing medicinal plant species used for the treatment of COVID-19, cough, and flu in Ogbomosho North and South Local Government Areas, Oyo State, Nigeria.

**Figure 6:**
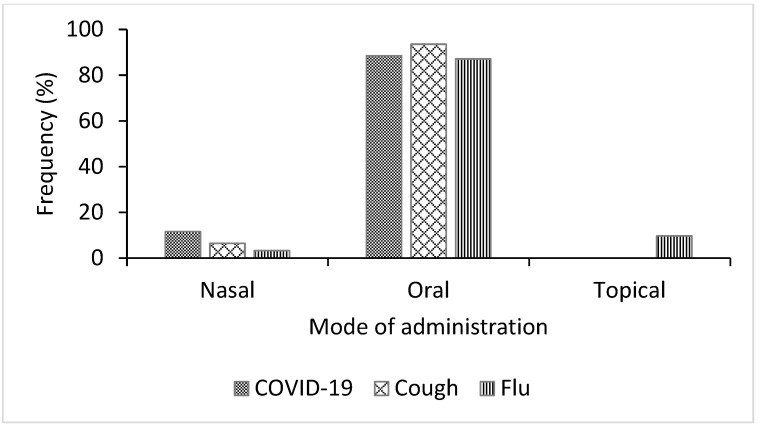
Mode of administration of medicinal plant species used for the treatment of COVID-19, cough, and flu in Ogbomosho North and South Local Government Areas, Oyo State, Nigeria.

While the participants in the study area identified 10 methods used for preparing the medicinal plant species, the majority of the herbal remedies were formulated by decoction for COVID-19 (81%) and by infusion for cough (28%) and flu (33%) (Figure 5). Several ethnobotanical studies have reported that decoction and infusion are the most cited methods of herbal remedy preparation [32,40,44,45]. Decoction and infusion are the most common forms of herbal remedy preparation in local communities, primarily because of the simplicity of the process [40]. Decoction involves heating the required quantity of the plant part in water for 30 min until about half of the water is lost [46], while the infusion is done by soaking the plant material in pre-warmed or cold water [44]. The herbal preparations in this study were administered orally, by snorting (nasal), and topically. However, the majority of the remedies were administered orally for COVID-19 (88%), cough (94%), and flu (87%) Figure 6. The dominance of oral administration may be explained by the fact that the oral route is simple and rapid and allows for better absorption of bioactive constituents in the medicinal plant [32].

### 3.5. Ethnobotanical Indices of Medicinal Plants Recorded

**Figure 7:**
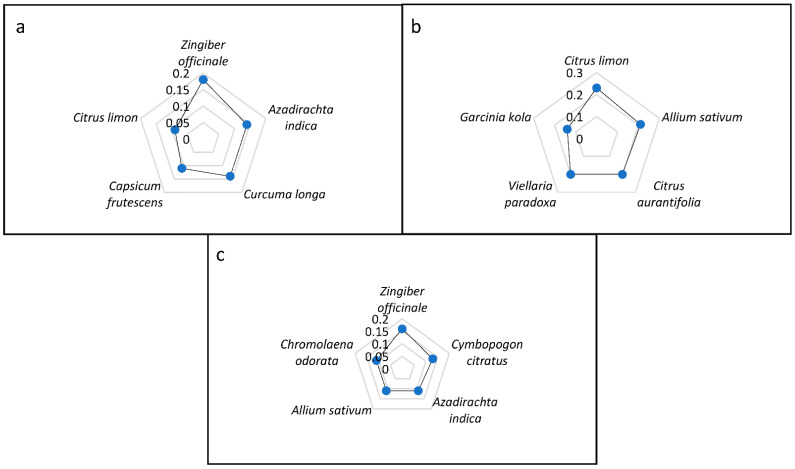
Relative frequency of citation of top five plant species used for the treatment of (**a**) COVID-19, (**b**) cough, and (**c**) flu in Ogbomosho North and South Local Government Areas, Oyo State, Nigeria.

Ethnobotanical indices are commonly used to deduce the local importance and relevance of medicinal plants in a given study area [45,47,48]. The indices can be used to rank medicinal plant species based on their acclaimed efficacy, cultural significance, and value. Additionally, these indices are valuable tools that give cues for further scientific investigation of medicinal plant species to discover their therapeutically bioactive constituents and for setting conservation and sustainable use plans [39]. In the present study, RF, RFC, and FL were used to determine the importance of the identified medicinal plant species used for treating and preventing COVID-19, cough, and flu in the study area (Table 2). The top five most cited species for treating COVID-19 were *Zingiber officinale* (FC = 10; RFC = 0.18; FL = 18%), *Curcuma longa* (FC = 8; RFC = 0.14; FL = 14%), *Azadirachta indica* (FC = 8; RFC = 0.14; FL = 14%), *Capsicum frutescens* (FC = 6; RFC = 0.11; FL = 11%), and *Citrus limon* (FC = 5; RFC = 0.09; FL = 9%). For cough, the top five cited species were *C. limon* (FC = 13; RFC = 0.23; FL = 23%), *Allium sativum* (FC = 12; RFC = 0.21; FL = 21%), *Citrus aurantiifolia* (FC = 11; RFC = 0.20; FL = 20%), *Vitellaria paradoxa* (FC = 11; RFC = 0.20; FL = 20%), and *Garcinia kola* (FC = 8; RFC = 0.14; FL = 14%). The top five most cited species for treating flu were *Z. officinale* (FC = 9; RFC = 0.16; FL = 16%), *Cymbopogon citratus* (FC = 7; RFC = 0.13; FL = 13%), *A. sativum* (FC = 6; RFC = 0.11; FL = 11%), *Chromolaena odorata* (FC = 6; RFC = 0.11; FL = 11%), and *A. indica* (FC = 6; RFC = 0.11; FL = 11%) (Figure 7a–c).

While ethnobotanical indices are claimed to be representations of the efficacy of medicinal plants and their potential use for drug development, it should be noted that these indices were not established by pharmacologists and statisticians [49]. Hence, the proof of concept is lacking. Additionally, the medicinal importance of plants and their cultural value cannot be summed up by numbers but are rather better obtained from a critical evaluation of the primary data based on the scope and objectives of the research [39,49].

The study’s most cited medicinal plants for treating COVID-19, including some major phytochemical components of these plants, have been reported to display antiviral and immunomodulatory activities, summarized in Table 5. For example, in an in silico docking study, Rajagopal et al. [50] reported that 8-gingerol and 10-gingerol isolated from *Z. officinale* were active against COVID-19 with significantly higher Glide scores when compared to hydroxychloroquine. Curcumin, a bioactive compound from *C. longa*, has been reported to exhibit antiviral activity against different types of enveloped viruses via several mechanisms such as induction of host antiviral responses, direct interaction with viral membrane proteins, and disruption of the viral envelope [13].

In a recent in vivo study, Supriyanto et al. [51] investigated the effect of the methanolic extract of *A. indica* leaf as an immunomodulator on different immune surveillance cells (CD4^+^, CD8^+^, CD25^+^, and CD62L). The results showed that *A. indica* demonstrated significant immunomodulatory activities against the cells by increasing pressure molecules and decreasing pro-inflammatory molecules. Capsaicin, the major bioactive component in *Capsicum* species, including *C. frutescens* identified in this study, has demonstrated antiviral activity and even structural disruption of viral 3CL-protease of COVID-19. Using molecular dynamics and strategies docking, Gonzalez-Paz et al. [52] evaluated the effect of capsaicin on viral 3CL-protease of COVID-19. The preliminary results from the study suggested that capsaicin can bind to the 3CL-protease of COVID-19, causing structural changes in the viral protease.

*C. limon*, one of the most cited species used in treating and preventing COVID-19 and cough in this study, is a well-known natural immune-modulator. Using different in silico and computational approaches, Khan et al. [53] investigated the effects of 25 phytochemicals isolated from *C. limon* against SARS-CoV-2 main protease (M^pro^), and their docking scores compared to remdesivir. The results revealed that six flavonoid compounds (diosmetin, quercetin, eriodictoyl, luteolin, spinacetin, and apigenin) exhibited good docking scores against SARS-CoV-2 M^pro^ without violating any drug-like activity standard parameters. Among these six compounds, diosmetin showed better docking values than the standard antiviral drug (remdesivir).

The promising antiviral and immunomodulatory activities displayed by some of the identified medicinal plants suggest that they are potential candidates for discovering new drugs in the fight against COVID-19 and related respiratory infections. Further in vivo and clinical studies should be done to evaluate their mechanism of action and antagonistic effects against COVID-19.

## 4. Conclusions

This ethnobotanical survey indicated high usage of medicinal remedies in Ogbomosho North and South Local Government Areas to prevent and treat COVID-19 and related respiratory infections, particularly cough and flu. Most plants documented for COVID-19 are also used to treat other respiratory tract infections, including the common cold. The phytochemical richness and biological activities (e.g., immunomodulatory, antiviral, antimicrobial, etc.) of some of the identified species have been documented. However, further studies on these plants’ phytochemical analysis and pharmacological potentials, particularly those with relatively high ethnobotanical indices, should be conducted. This will help identify bioactive constituents and inform potential drug development to treat respiratory ailments, including COVID-19. Although the increasing global demand for herbal medicine is attributed to the belief that natural products (in this case, medicinal plants) are safe, toxicity studies should be done on the documented species to assess and establish their safety for human use. It is noteworthy that the increasing exploitation of medicinal plants for different uses might endanger the species. Hence, the conservation of these medicinal species should be prioritized for continual and sustainable use.

## Figures and Tables

**Table 1:** Demographic information of participants (n = 56) in the study area.

Parameters	Group	Number	Percentage (%)
Gender	Male	23	41.1
	Female	33	58.9
Age (Years)	20–30	4	7.14
	31–40	7	12.5
	41–50	14	25
	51 and above	31	55.4
Local Government Area	Ogbomosho South	41	73.21
	Ogbomosho North	16	26.79
Educational Level	Primary	26	46.43
	Secondary	19	33.93
	Tertiary	11	19.64
Occupation	Herb seller	27	48.2
	Traditional health practitioner	6	10.7
	Farmer	1	1.8
	Other	22	39.3

**Table 2:** Medicinal plants used to treat and prevent COVID-19 in Ogbomosho North and South Local Government Areas, Oyo State, Nigeria.

S/N	Scientific Name and Voucher Specimen Number	Common Name	Local Name	Family	Mode of Preparation	Growth Form	Mode of Administration	Plant Part	Plant Source	Material	FC	RFC	FL (%)
1	*Allium sativum* L. IFE18082	Garlic	Ayu	Amaryllidaceae	Frying	Herb	Nasal	Bulbs	C	F	3	0.05	5
2	*Alstonia boonei* De Wild. IFE18083	Stool weed	Doctor igbo	Apocynaceae	Decoction	Tree	Oral	Leaves	C	F	1	0.02	2
3	*Azadirachta indica* A. Juss. IFE18086	Neem	Dongoyaro	Meliaceae	Decoction	Tree	Oral	Leaves, bark	WP	F	8	0.14	14
4	*Capsicum frutescens* L. IFE18088	Pepper	Ata wewe	Solanaceae	Decoction	Herb	Oral	Fruit	C	F	6	0.11	11
5	*Cinnamomum camphora* (L.) J. Presl IFE18091	Camphor	Kafura	Lauraceae	Heating	Tree	Nasal	Bark	WP	F	1	0.02	2
6	*Citrus limon* (L.) Osbeck IFE18093	Lime	Oronbo	Rutaceae	Juice extraction	Tree	Oral	Fruit	C	F	5	0.014	9
7	*Clausena anisata* (Willd.) Hook.f. ex Benth. IFE18094	Horsewood	Agbasa	Rutaceae	Decoction	Tree	Oral	Leaves	WP	F	1	0.02	2
8	*Curcuma longa* L. IFE18134	Turmeric	Ata ile pupa	Zingiberaceae	Decoction	Herb	Oral	Rhizomes	C	D	8	0.14	14
9	*Cymbopogon citratus* (DC.) Stapf IFE18097	Lemon Grass	Ewe tea	Poaceae	Decoction	Grass	Oral	Leaves	C	F	1	0.02	2
10	*Drypetes gossweileri* S.Moore IFE18099	Horse radish Tree	Epo aganwo	Putranjivaceae	Decoction	Tree	Oral	Bark	WP	D	1	0.02	2
11	*Gymnanthemum amygdalinum* (Delile) Sch.Bip. IFE18127	Bitter leaf	Ewuro	Asteraceae	Decoction	Shrub	Oral	Leaves	C	F	2	0.04	4
12	*Momordica charantia* L. IFE18108	Bitter lemon	Ejinrin	Cucurbitaceae	Decoction	Climber	Oral	Leaves	C	F	1	0.02	2
13	*Morinda lucida* Benth. IFE18109	Brimstone Tree	Oruwo	Rubiaceae	Decoction	Tree	Oral	Leaves, bark	WP	D	3	0.05	5
14	*Neonauclea excelsa* (Blume) Merr. IFE18111	Nauclea	Egbeesi	Rubiaceae	Decoction	Tree	Oral	Bark	WP	F	1	0.02	2
15	*Nigella sativa* L. IFE18113	Black seed	Asofeyeje	Ranunculaceae	Decoction	Herb	Oral	Seeds	WP	D	1	0.02	2
16	*Peperomia pellucida* (L.) Kunth IFE18115	Shiny bush	Ewe rinrin	Piperaceae	Pulverizing	Herb	Oral	Leaves	C	D	1	0.02	2
17	*Pseudocedrela kotschyi* Harms IFE18117	Dry zone cedar	Emigbegi	Meliaceae	Decoction	Tree	Oral	Leaves	WP	F	1	0.02	2
18	*Senna alata* (L.) Roxb. IFE18121	Candle bush	Asunwon oyinbo	Fabaceae	Decoction	Tree	Oral	Leaves	WP	F	2	0.04	4
19	*Tetrapleura tetraptera* (Schum. and Thonn.) Taub. IFE18123	Aidan Tree	Aridan	Fabaceae	Decoction	Tree	Oral	Fruit	C	F	3	0.05	5
20	*Tithonia diversifolia* (Hemsl.) A.Gray IFE18124	Tree marigold	Sepeleba	Asteraceae	Decoction	Herb	Oral	Leaves	WP	F	1	0.02	2
21	*Uvaria afzelii* Scott. Elliot IFE18125	Monkey finger	Gbogbonise	Annonaceae	Decoction	Tree	Oral	Bark	WP	D	1	0.02	2
22	*Uvaria chamae* P. Beauv. IFE18126	Finger root	Eruiju	Annonaceae	Decoction	Herb	Oral	Bark	C	F	1	0.02	2
23	*Vitellaria paradoxa* C.F Gaertn IFE18128	Shea butter	Ori	Sapotaceae	Heating	Tree	Nasal	Seeds	WP	D	6	0.11	11
24	*Xylopia villosa* Chipp IFE18130	Black palufon	Eeru awonka	Annonaceae	Decoction	Tree	Oral	Leaves	WP	F	1	0.02	2
25	*Zea mays* L. IFE18131	Maize	Agbado	Poaceae	Decoction	Grass	Oral	Seeds	C	F	1	0.02	2
26	*Zingiber officinale* Roscoe IFE18132	Ginger	Ata ile	Zingiberaceae	Decoction	Herb	Oral	Rhizomes	C	F	10	0.18	18

Plant source (C—cultivated; WP—wild populations); Material (F—fresh; D—dried); FC—frequency of citation; RFC—relative frequency of citation; FL—fidelity level.

**Table 3:** Medicinal plants used to treat and prevent cough in Ogbomosho North and South Local Government Areas, Oyo State, Nigeria.

S/N	Scientific Name and Voucher Specimen Number	Common Name	Local Name	Family	Mode of Preparation	Growth Form	Mode of Administration	Plant Part	Plant Source	Material	FC	RFC	FL (%)
1	*Abrus precatorius* L. IFE18077	Rosary pea	Omisinmisin	Fabaceae	Decoction	Climber	Oral	Leaves	WP	F	7	0.13	13
2	*Aframomum melegueta* K. Schum. IFE18079	Alligator pepper	Ataare	Zingiberaceae	Pulverizing	Herb	Oral	Seeds, fruit	C	D	5	0.09	9
3	*Allium ascalonicum* L. IFE18080	Spring onion	Alubosa elewe	Amaryllidaceae	Grating, Infusion	Herb	Oral	Bulbs	C	F	1	0.02	2
4	*Allium cepa* L. IFE18081	Onion	Alubosa	Amaryllidaceae	Pulverizing	Shrub	Oral	Bulbs	C	F	3	0.05	5
5	*Allium sativum* L. IFE18082	Garlic	Ayu	Amaryllidaceae	Frying	Herb	Nasal	Bulbs	C	F	12	0.21	21
6	*Amaranthus spinosus* L. IFE18084	Spinach	Igi teteregun	Amaranthaceae	Decoction	Herb	Oral	Bark	C	F	2	0.04	4
7	*Capsicum frutescens* L. IFE18088	Hot pepper	Ata wewe	Solanaceae	Pulverizing, Infusion	Herb	Oral	Seeds	C	D	2	0.04	4
8	*Citrus aurantiifolia* (Christm.) Swingle IFE18092	Lemon	Osan wewe	Rutaceae	Juice extraction	Tree	Oral	Fruit	C	F	11	0.2	20
9	*Citrus limon* (L.) Osbeck IFE18093	Lime	Orombo	Rutaceae	Juice extraction	Tree	Oral	Fruit	C	F	13	0.23	23
10	*Cocos nucifera* L. IFE18095	Coconut	Agbon	Arecaceae	Decoction	Tree	Oral	Pod	C	F	2	0.04	4
11	*Crinum jagus* (J.Thomps.) Dandy IFE18096	Poison bulb	Ogede odo	Amaryllidaceae	Pulverizing, grating, Infusion	Herb	Oral	Bulbs	WP	F	2	0.04	4
12	*Cymbopogon citratus* Stapf IFE18097	Lemon Grass	Ewe tea	Poaceae	Decoction	Grass	Oral	Leaves	C	F	2	0.04	4
13	*Elaeis guineensis* Jacq. IFE18100	Palm Tree	Ope	Arecaceae	Infusion	Tree	Oral	Seeds	C	F	2	0.04	4
14	*Eucalyptus globulus* Labill IFE18101	Bluegum Eucalyptus	Eucalyptus	Myrtaceae	Decoction	Tree	Oral	Leaves	WP	F	2	0.04	4
15	*Ficus asperifolia* Hook. ex Miq. IFE18103	Sandpaper	Ewe ipin	Moraceae	Infusion	Tree	Oral	Leaves	WP	D	2	0.04	4
16	*Garcinia kola* Heckel IFE18105	Bitter cola	Orogbo	Clusiaceae	Infusion, pulverizing	Tree	Oral	Seeds	C	F	8	0.14	14
17	*Gymnanthemum amygdalinum* (Delile) Sch.Bip. IFE18127	Bitter leaf	Ewuro	Asteraceae	Pulverizing, Infusion	Shrub	Oral	Flower	C	F	4	0.07	7
18	*Jatropha curcas* L. IFE18106	Jatropha	Lapalapa	Euphorbiaceae	Decoction	Shrub	Oral	Fruit	WP	F	4	0.07	7
19	*Mangifera indica* L. IFE18107	Mango	Mangoro	Anacardiaceae	Decoction	Tree	Oral	Bark	C	F	2	0.04	4
20	*Marsdenia latifolia* (Benth.) K.Schum. IFE18104	Bush buck	Arokeke, Madunmaro	Apocynaceae	Maceration, infusion	Shrub	Oral	Leaves, bark	WP	F	1	0.02	2
21	*Neonauclea excelsa* (Blume) Merr. IFE18111	Nauclea	Egbeesi	Rubiaceae	Infusion	Tree	Oral	Root	WP	F	5	0.09	9
22	*Olax subscorpioidea* Oliv. IFE18114	Ivory coast	Ifon	Olacaceae	Pulverizing	Tree	Oral	Bark	WP	D	2	0.04	4
23	*Psidium guajava* L. IFE18118	Guava	Groofa	Myrtaceae	Decoction	Tree	Oral	Bark, Leaves	C	F	3	0.05	5
24	*Saccharum officinarum L.* IFE18119	Sugarcane	Ireke	Poaceae	Pounding, Infusion	Grass	Oral	Stem	C	F	1	0.02	2
25	*Securidaca longipedunculata* Fresen. IFE18120	Violet Tree	Ipeta	Polygalaceae	Pulverizing	Tree	Oral	Bark	WP	D	3	0.05	5
26	*Spondias mombin* L. IFE18122	Hog plum	Iyeye	Anacardiaceae	Decoction	Tree	Oral	Bark	WP	F	5	0.09	9
27	*Tetrapleura tetraptera* (Schum. and Thonn.) Taub. IFE18123	Aidan Tree	Aridan/ Aidan	Fabaceae	Pulverizing, grating, Infusion	Tree	Oral	Fruit	C	F	1	0.02	2
28	*Vitellaria paradoxa* C.F Gaertn IFE18128	Shea butter	Ori	Sapotaceae	Frying	Tree	Nasal	Seeds	WP	D	11	0.2	20
29	*Xylopia aethiopica* A. Rich IFE18129	African pepper	Eeru	Annonaceae	Pulverizing, Infusion	Tree	Oral	Seeds	WP	D	6	0.11	11
30	*Zea mays* L. IFE18131	Maize	Agbado	Poaceae	Decoction	Grass	Oral	Husk	C	F	2	0.04	4
31	*Zingiber officinale* Roscoe IFE18132	Ginger	Ata ile	Zingiberaceae	Decoction, pulverizing	Herb	Oral	Rhizomes, root	C	F	3	0.05	5

Plant source (C—cultivated; WP—wild populations); Material (F—fresh; D—dried); FC—frequency of citation; RFC—relative frequency of citation; FL—fidelity level.

**Table 4:** Medicinal plants used to treat and prevent flu in Ogbomosho North and South Local Government Areas, Oyo State, Nigeria.

S/N	Scientific Name and Voucher Specimen Number	Common Name	Local Name	Family	Mode of Preparation	Growth Form	Mode of Administration	Plant Part	Plant Source	Material	FC	RFC	FL (%)
1	*Aframomum melegueta* K. Schum. IFE18079	Alligator pepper	Ataare	Zingiberaceae	Decoction	Herb	Oral	Seeds	C	D	2	0.04	4
2	*Allium ascalonicum* L. IFE18080	Spring onion	Alubosa elewe	Amaryllidaceae	Juice extraction	Herb	Oral	Bulbs	C	F	2	0.04	4
3	*Allium cepa* L. IFE18081	White onion	Alubosa funfun	Amaryllidaceae	Infusion	Shrub	Oral	Bulbs	C	F	2	0.04	4
4	*Allium sativum* L. IFE18082	Garlic	Ayu	Amaryllidaceae	Chewing, frying	Herb	Oral, nasal	Bulbs	C	F	6	0.11	11
5	*Annona senegalensis* Pers. IFE18085	Wild soursop	Ewe abo	Annonaceae	Decoction	Shrub	Oral	Leaves	WP	F	2	0.04	4
6	*Azadirachta indica* A. Juss IFE18086	Neem	Dongoyaro	Meliaceae	Decoction, infusion	Tree	Oral	Leaves, bark	WP	F	6	0.11	11
7	*Capsicum annuum* L. IFE18087	Hot pepper	Ata ijoosi	Solanaceae	Pulverizing	Herb	Oral	Seeds	C	D	2	0.04	4
8	*Carica papaya* L. IFE18089	Pawpaw	Eso ibepe	Caricaceae	Decoction	Tree	Oral	Seeds	C	F	3	0.05	5
9	*Chromolaena odorata* (L.) R.M.King and H.Rob. IFE18090	Siam weed	Ewe akintola	Asteraceae	Pulverizing, infusion	Shrub	Oral	Leaves	C	F,D	6	0.11	11
10	*Cinnamomum camphora* (L.) J.Presl IFE18091	Camphor	Kafura	Lauraceae	Frying	Tree	Topical	Bark	WP	F	1	0.02	2
11	*Citrus limon* (L.) Osbeck IFE18093	Lime	Oronbo	Rutaceae	Juice extraction	Tree	Topical	Fruit	C	F	3	0.05	5
12	*Crinum jagus* (J.Thomps.) Dandy IFE18096	Poison bulb	Ogede odo	Amaryllidaceae	Pounding	Herb	Oral	Bulbs	WP	F	2	0.04	4
13	*Cymbopogon citratus* (DC.) Stapf IFE18097	Lemon Grass	Ewe tea	Poaceae	Decoction, infusion	Grass	Oral	Leaves	C	F	7	0.13	13
14	*Eucalyptus globulus* LabillIFE18101	Bluegum Eucalyptus	Eucalyptus	Myrtaceae	Juice extraction	Tree	Oral	Leaves	WP	F	1	0.02	2
15	*Garcinia kola* Heckel IFE18105	Bitter cola	Orogbo	Clusiaceae	Pulverizing	Tree	Oral	Seeds	C	F	5	0.09	9
16	*Gymnanthemum amygdalinum* (Delile) Sch.Bip IFE18127	Bitter leaf	Ewuro	Asteraceae	Pulverizing, infusion	Shrub	Oral	Leaves	C	F	4	0.07	7
17	*Kigelia africana* (Lam.) Benth. IFE18133	Sausage Tree	Pandoro	Bignoniaceae	Pulverizing, infusion	Tree	Oral	Bark	WP	D	2	0.04	4
18	*Mangifera indica* L. IFE18107	Mango	Mango	Anacardiaceae	Infusion	Tree	Oral	Leaves	C	F	2	0.04	4
19	*Morinda lucida* Benth. IFE18109	Brimstone Tree	Oruwo	Rubiaceae	Pulverizing, infusion	Tree	Oral	Bark	WP	D	2	0.04	4
20	*Musa × paradisiaca* L. IFE18110	Banana	Ogede	Musaceae	Juice extraction	Herb	Oral	Leaves	C	F	1	0.02	2
21	*Nicotiana tabacum* L. IFE18112	Tobacco	Taba	Solanaceae	Infusion	Herb	Oral	Leaves	C	F	1	0.02	2
22	*Piper nigrum* L. IFE18116	Black pepper	Iyere	Piperaceae	Decoction	Climber	Oral	Seeds	C	D	1	0.02	2
23	*Syzygium aromaticum* (L.) Merr. and L.M. Perry IFE18102	Cloves	kannafuru	Myrtaceae	Infusion, pulverizing	Tree	Oral	Seeds	C	D, F	1	0.02	2
24	*Tetrapleura tetraptera* (Schum. and Thonn.) Taub. IFE18123	Aidan Tree	Aridan	Fabaceae	Decoction, infusion, pulverizing	Tree	Oral	Fruit	C	D, F	1	0.02	2
25	*Uvariopsis tripetala* (Baker f.) G.E.Schatz IFE18098	Pepper fruit	Ata dudu, ata igbere	Annonaceae	Decoction	Tree	Oral	Seeds	WP	D	1	0.02	2
26	*Vachellia nilotica* (L.) P.J.H.Hurter and Mabb. IFE18078	Gum Arabic Tree	Booni	Fabaceae	Pulverizing, infusion	Tree	Oral	Seeds	WP	D, F	1	0.02	2
27	*Vitellaria paradoxa* C.F.Gaertn IFE18128	Shea butter	Ori	Sapotaceae	Frying	Tree	Topical	Seeds	WP	D	1	0.02	2
28	*Xylopia aethiopica* A. Rich. IFE18129	African pepper	Eeru	Annonaceae	Pulverizing, infusion, decoction	Tree	Oral	Seeds	WP	D	2	0.04	4
29	*Zingiber officinale* Roscoe IFE18132	Ginger	Ata ile	Zingiberaceae	Pulverizing, infusion	Herb	Oral	Root	C	F	9	0.16	16

Plant source (C—cultivated; WP—wild populations); Material (F—fresh; D—dried); FC—frequency of citation; RFC—relative frequency of citation; FL—fidelity level.

**Table 5:** Antiviral and immunomodulatory properties of top-cited species used for the treatment of COVID-19, cough, and flu.

Species	Antiviral Activities	Immunomodulatory Activities	Active Compounds	Mechanism of Action	References
*Allium sativum* L.	Clinical study revealed that allicin–garlic capsule prevented common cold in active treatment group than the placebo group.	In vitro study showed that polysaccharides isolated from fresh garlic promoted immune functions of RAW 264.7 macrophages	Allicin	Antiviral effects of allicin by enhancing immune response. Immunomodulatory effect by promotion of phagocytosis, release of NO, and expressions of several immune-related cytokines.	[54,55,56]
*Azadirachta indica* A.Juss.	In vivo study showed that aqueous *A. indica* leaf extract significantly decreased HCV seropositivity and inhibited the replication of HCV.	In vitro study showed that ethanol extract of *A. indica* downregulated the levels of CD_4_ + T cell activation, and inhibited SEB induced CD_4_+ T-cell activation/exhaustion	Azadirachtin 3-Deacetyl-3-azadirachtin	Antiviral effects by binding to NS3 protease of HCV.	[57,58,59]
*Capsicum frutescens* L.	Molecular dynamics and strategies docking study showed that capsaicin caused structural disruption of viral 3CL-protease of COVID-19.	Capsaicin attenuated chronic stress-induced immunosuppression in BALB/c mice.	Capsaicin	Antiviral effect by binding to 3CL-protease of COVID-19. Immunomodulatory activity through increased production of Th1 cytokines and decreased production of Th2 cytokines and TGF-β1.	[53,60]
*Chromolaena odorata* (L.) R.M.King and H.Rob.	NR	Ethanolic extract of *C. odorata* showed immunopotentiating activities on the innate immunity of Balb/C mice, and reverse a drug-induced immunosuppression. In another report, soluble polysaccharides (PoS) fraction of *C. odorata* showed immunostimulatory activity.	Polysaccharides	Immunostimulatory activity of PoS fraction via stimulation of peripheral blood mononuclear cells, and production of IFN-γ.	[61,62]
*Citrus aurantiifolia* (Christm.) Swingle	Limonene showed moderate inhibition of the avian influenza A virus (H5N1).	Limonene showed immunoregulatory activity in lipopolysaccharides (LPS)-induced pleurisy model.	Limonene	Antiviral effects of limonene by the inhibition of viral replication via direct action on the virus. Immunomodulatory effects by the inhibition of NO and the cytokines IFN-γ and IL-4.	[63,64,65]
*Citrus limon* (L.) Osbeck	In vitro study showed that limonin reduced the replication of New-castle disease virus (NDV) in all cell lines.	Ethanolic extract of lemon peel increased proliferation of mouse splenocytes signifying immunostimulation activity	Limonin	Antiviral activity of limoin by downregulation of NDV- haemagglutinin-neuraminidase and matrix genes. Immunomodulatory effect by augmentation of proliferation of T-lymphocytes.	[66,67]
*Curcuma longa* L.	In vitro study showed that curcumin directly inactivated influenza A virus (IAV) blocked IAV adsorption, and inhibited IAV proliferation.	Poly D,L-lactic-co-glycolic acid entrapped curcumin nanoparticle significantly stimulated primary humoral immune response in mice.	Curcumin	Antiviral effects by Increasing Nrf2, HO-1, NQO1, GSTA3 and IFN-β production; suppression of IAV-induced activation of TLR2/4/7, Akt, p38/JNK MAPK and NF-κB pathways.	[68,69]
*Cymbopogon citratus* (DC.) Stapf	In vitro and in silico studies showed that the methanolic extract of *C. citratus* demonstrated anti-dengue activities. Additionally, E-Citral, Z-Citral, and β-Myrcene from the essential oil of *C. citratus* showed significant inhibitory effect on herpes simplex virus-1 (HSV-1) replication.	Oral citral administration showed marked immunomodulatory effects in lipopolysaccharides (LPS)- induced paw edema in mice.	Citral	Immunomodulatory effects by the inhibition of oxidative activity, activation of nuclear factor kappa B, peroxisome proliferator-activated receptor (PPAR)-α and γ.	[70,71,72]
*Garcinia kola* Heckel	Kolaviron, extracted from *G. kola* seeds impaired IAV-induced mortality and was effective in delaying the clinical symptoms of IAV in BALB/c mice.	In vivo and in vitro studies revealed that kolaviron demonstrated immunomodulatory and immunorestorative properties in immunocompetent and immunocompromised animal models.	Kolaviron	Antiviral effects by attenuating nitric oxide production and suppression of myeloperoxidase activity, immunomodulatory effect by inhibition of delayed-type hypersensitivity response and enhancement of leukocytes counts.	[73,74,75]
*Vitellaria paradoxa* C.F.Gaertn.	Ethanolic extract of *V. paradoxa* inhibited 50% of human polio virus (Type 1) and Astrovirus.	Methnolic extract of *V. paradoxa* displayed significant suppression of inflammation response in carrageenan-induced inflammation in Wistar albino rats.	Catechins	Inflammation suppression effects by the inhibition of the pro-inflammatory cytokines IL-1, IL-6, and TNF-α.	[76,77]
*Zingiber officinale* Roscoe	In a clinical study, ethanolic extract of *Z. officinale* showed significant decrease in the viral load of patients with hepatitis C virus (HCV). In a recent in silico docking study, 8-gingerol and 10-gingerol isolated from *Z. officinale* were active against COVID-19 with significantly higher Glide scores when compared to hydroxychloroquine.	Neutral ginger polysaccharide fraction (NGP) significantly showed immunomodulatory activity on RAW264.7 cells without cytotoxicity,	Gingerol Neutral ginger polysaccharide	Immunomodulatory effects by the promotion of TNF-α production by macrophage cells.	[78,79,80]

NR—Not reported.

## Data Availability

Not applicable.
